# Application of Ensemble Machine Learning Methods to Estimate the Compressive Strength of Fiber-Reinforced Nano-Silica Modified Concrete

**DOI:** 10.3390/polym14183906

**Published:** 2022-09-19

**Authors:** Madiha Anjum, Kaffayatullah Khan, Waqas Ahmad, Ayaz Ahmad, Muhammad Nasir Amin, Afnan Nafees

**Affiliations:** 1 Department of Computer Engineering, College of Computer Science and Information, Technology, King Faisal University, Al-Ahsa 31982, Saudi Arabia; 2 Department of Civil and Environmental Engineering, College of Engineering, King Faisal University, Al-Ahsa 31982, Saudi Arabia; 3 Department of Civil Engineering, COMSATS University Islamabad, Abbottabad 22060, Pakistan; 4 MaREI Centre, Ryan Institute and School of Engineering, College of Science and Engineering, National University of Ireland Galway, H91 TK33 Galway, Ireland

**Keywords:** concrete, fiber-reinforced concrete, nano-silica, nano-silica modified concrete, compressive strength

## Abstract

In this study, compressive strength (CS) of fiber-reinforced nano-silica concrete (FRNSC) was anticipated using ensemble machine learning (ML) approaches. Four types of ensemble ML methods were employed, including gradient boosting, random forest, bagging regressor, and AdaBoost regressor, to achieve the study’s aims. The validity of employed models was tested and compared using the statistical tests, coefficient of determination (R^2^), and k-fold method. Moreover, a Shapley Additive Explanations (SHAP) analysis was used to observe the interaction and effect of input parameters on the CS of FRNSC. Six input features, including fiber volume, coarse aggregate to fine aggregate ratio, water to binder ratio, nano-silica, superplasticizer to binder ratio, and specimen age, were used for modeling. In predicting the CS of FRNSC, it was observed that gradient boosting was the model of lower accuracy and the AdaBoost regressor had the highest precision in forecasting the CS of FRNSC. However, the performance of random forest and the bagging regressor was also comparable to that of the AdaBoost regressor model. The R^2^ for the gradient boosting, random forest, bagging regressor, and AdaBoost regressor models were 0.82, 0.91, 0.91, and 0.92, respectively. Also, the error values of the models further validated the exactness of the ML methods. The average error values for the gradient boosting, random forest, bagging regressor, and AdaBoost regressor models were 5.92, 4.38, 4.24, and 3.73 MPa, respectively. SHAP study discovered that the coarse aggregate to fine aggregate ratio shows a greater negative correlation with FRNSC’s CS. However, specimen age affects FRNSC CS positively. Nano-silica, fiber volume, and the ratio of superplasticizer to binder have both positive and deleterious effects on the CS of FRNSC. Employing these methods will promote the building sector by presenting fast and economical methods for calculating material properties and the impact of raw ingredients.

## 1. Introduction

Concrete is an extremely popular building material [1,2,3,4,5]. To reduce the brittle behavior of concrete, researchers have been interested in fiber-reinforced concrete (FRC), which has much more ductility than standard concrete [6,7,8,9,10]. Concrete failure begins with the appearance of cracks. The purpose of introducing FRC, composed of several fibers like glass, steel, and polypropylene, is to enhance the mechanical performance and energy absorption capability of concrete by restricting the crack growth so that the structural elements can endure greater distortions following the development of the early cracks [11,12,13,14,15]. Nanoparticles, such as nano-silica (NS), have been proven to fill the voids of cement paste and increase the durability and mechanical performance of concrete [16,17,18,19]. Hence, the usage of nanoparticles in FRC might result in a material with better performance that is ideal for the building of long-lasting, high-performance structures. The initial and final setting times of the concrete were decreased by NS, and its early age strength was enhanced. An essential aspect of NS is its nanostructure, which offers an extraordinarily larger specific surface area (SSA) and thus functions as a cement-aggregate binder [20]. Nanoparticle size is responsible for NS’s strong pozzolanic action [21,22]. The interfacial transition zone (ITZ), which is considered to be a weak point in concrete, is also enhanced [23] due to the fact that these nanoparticles fill all gaps and voids [24], hence reducing permeability. It has been demonstrated that NS is a very effective element that speeds up the hydration process of concrete [25] and creates more calcium-silicate-hydrate (C-S-H) gel [26,27], which is responsible for the strength of a material [28,29]. In cementitious materials, the fraction of portlandite-Ca(OH)_2_ reduces as NS mixes with Ca(OH)_2_ to produce a denser product [30]. Certain earlier research indicates that replacing NS for up to 4% of the cement can increase its durability and strength under unfavorable conditions like corrosion and high temperatures [31,32]. Although several research studies have proven the usage of NS for specific applications of concretes, it has been found to be extremely effective when used at a percentage of 0.5 to 4% as a cement replacement. The excessive quantity of NS may result in particle accumulation due to non-uniform dispersal, thus reducing workability [33]. Numerous nanoparticles are used as additives in concrete to enhance their macroscopic properties and performance, and NS has become commonplace amongst these nanoparticles. Nonetheless, the limited practical applications of NS in the building are due to their higher expenses, which are roughly 1000 times more costly than regular cement [34,35].

Several experiments are conducted to evaluate the concrete performance, but compressive strength (CS) is commonly considered extremely critical [36]. CS of concrete provides valuable information about its many properties. Concrete’s CS is directly or indirectly linked to a range of mechanical and durability characteristics [37]. To reduce unnecessary experimentation and resource waste, forecasting models for the strength of materials are now being established. Numerous standard models, including best-fit curves, are used to mimic the characteristics of concrete (formed on regression analysis). Due to the nonlinear nature of cement-based composites [6,38], regression approaches developed in this approach may not effectively signify the material’s fundamental performance. Moreover, regression methods might exaggerate the significance of particular factors [39]. Methods based on artificial intelligence (AI), such as supervised machine learning (ML), are amongst the highly innovative modeling techniques employed in the current subject domain [40,41,42,43,44]. These approaches model responses utilizing input features, and the resultant models are backed by testing. ML approaches are used to predict the features of concrete and bituminous mixes [45,46,47,48,49,50,51,52]. 

In addition to experimental research, the application of various ML algorithms to forecast the fresh and hardened characteristics of concrete mixes has been proven to provide considerable benefits [53,54]. Using AI technology, a previous study suggested a new data-driven formulation for estimating the CS of foam cellular concrete. The generated model can estimate CS and beats all empirical models [55]. Other similar studies explored the capability of an AI system to forecast the characteristics of concrete. The AI technique was suggested as an alternative to an experimental program for modeling the fresh and hardened characteristics of concrete [56,57]. Behnood et al. [58] examined the model tree as an AI technique for predicting the CS of separate data records of normal and high-performance concrete. They discovered that the model tree-based categorization technique might provide very accurate prediction formulas. Gholampour et al. [59] concentrated on the application of AI techniques for estimating the mechanical parameters of recycled aggregate concrete. This research determined the applicability of predictive AI models in pre-design and modeling. It was determined that most of the earlier ML-based investigations concentrated on forecasting the CS of normal cement-based materials [60,61,62,63,64,65,66]; just a few papers focused on predicting the properties of fiber-reinforced nano-silica concrete (FRNSC).

This study concentrates on ML methods utilization to calculate the CS of FRNSC. Four types of ensemble ML methods were employed, including gradient boosting (GB), random forest (RF), bagging regressor (BR), and AdaBoost regressor (AR), to achieve the study’s aims. The performance of each model was assessed using statistical tests, coefficients of determination (R^2^), k-fold method, and variance of projected findings (errors) from those of actual. The reason for choosing only ensemble ML methods is because it is evident from the literature that ensemble ML methods outperform individual ML methods [67,68]. Therefore, this study employed only ensemble ML methods to assess which one is the best predictor. Though experimental investigations need substantial human effort, expenditures, and time for materials gathering, casting, curing, and performing tests, by overcoming the aforesaid difficulties through the application of innovative methodologies such as ML, the building sector will acquire an edge. Since a variety of variables, such as fiber volume, the aggregate amount, water to binder ratio, nano-silica dosage, etc., impact the concrete CS, it is challenging to evaluate their combined influence using experimental approaches. In this context, a Shapley Additive Explanations (SHAP) analysis was performed to explore the interaction and influence of input parameters on the CS of FRNSC. A data set is necessary for ML techniques and SHAP analysis, which may be gathered from past studies as different experimental research has been done to demonstrate the CS of FRNSC. The obtained data might then be employed to train ML systems and estimate material properties. The current research utilized six input parameters and 175 data samples to forecast the CS of FRNSC and assess the performance of multiple ML techniques. The objective of this work is to determine the most suited ML approach for predicting the CS of GPC and the influence of many factors on FRNSC strength.

## 2. Research Strategy

### 2.1. Dataset Description

**Figure 1:**
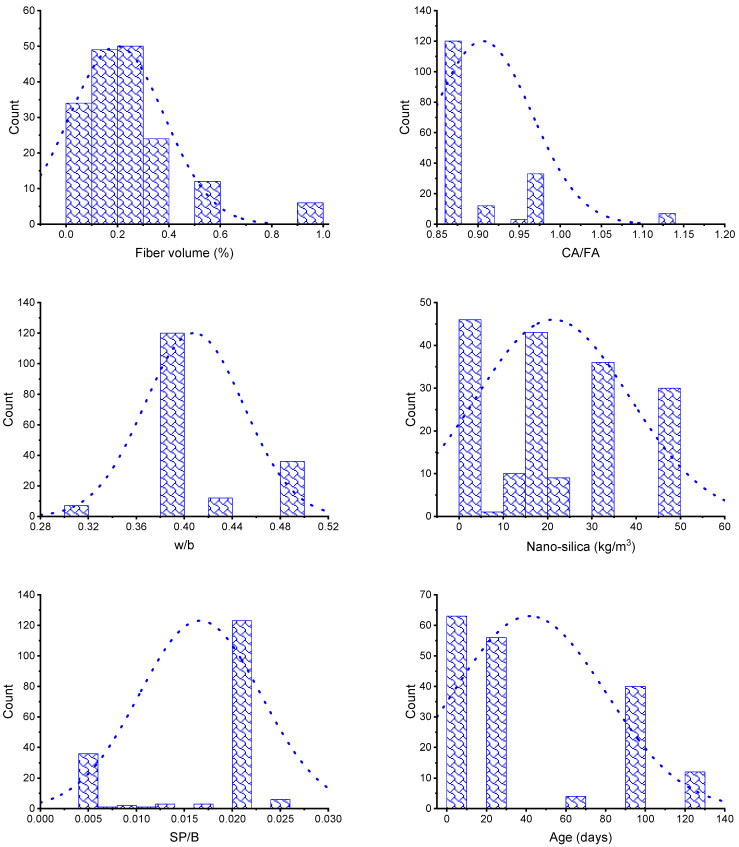
Input parameter’s relative frequency dispersion.

In order to develop the required result, ML methods need a vast diversity of input variables [69]. The CS of FRNSC was computed using literature data (see Supplementary Data). To avoid bias, data samples were collected arbitrarily from previous studies, and data points containing CS results were collected for algorithm execution. Fiber volume (FV), coarse aggregate to fine aggregate ratio (CA/FA), water to binder ratio (w/b), nano-silica (NS), superplasticizer to binder ratio (SP/B), and specimen age (A) were incorporated as inputs in the models, with CS acting as an output. The amount of input features and size of the dataset have a significant impact on the model’s output [70]. In this research, 175 data samples were employed to run ML models. Three kinds of fibers were utilized in the FRNSC samples, including steel, polypropylene, and glass fibers. The data were obtained based on the proportions of the mixture and the desired result in a concern, as models required comparable types of input parameters for each mixture to yield the required output. The descriptive statistics for each input variable are given in Table 1. The word “descriptive statistics” indicates a collection of brief, scientific measurements that give an outcome, which might be the entire population or its subgroup. The mode, median, and mean reveal basic trends, whereas the standard deviation, minimum, and maximum indicate variance. Table 1 comprises all statistical terms for the input variables. Figure 1 depicts the relative frequency dispersal of each input component.

### 2.2. Modeling

**Figure 2:**
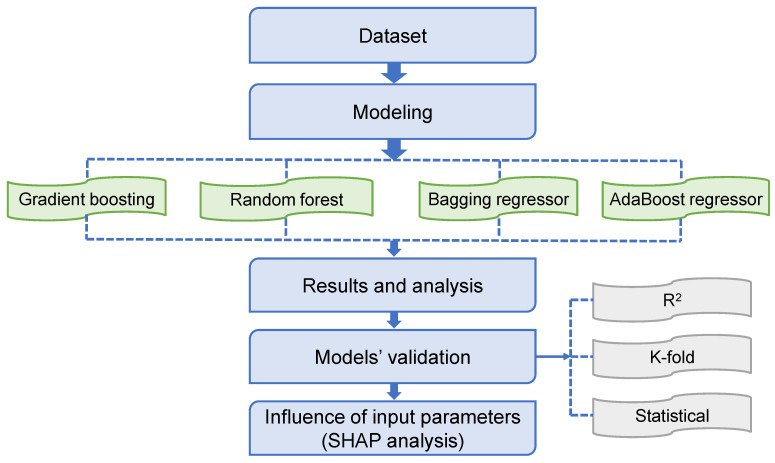
Sequence of the research strategy adopted.

To meet the study’s aims, four ensemble ML methods, including GB, RF, BR, and AR, were used with Python coding employing the Anaconda Navigator software. Spyder (5.1.5) was utilized to execute the ML models. In the presence of input variables, these ML approaches are frequently employed to estimate the intended output. These approaches may forecast the temperature effects, the strength properties, and the durability of the material [71,72]. During the modeling stage, six input characteristics and one output (CS) were used. The R^2^ value of the expected outcome represents the performing ability of the applied techniques. The R^2^ value defines the extent of deviation; a number close to zero signifies larger variation, whilst a value close to one implies that the predicted model and actual data are almost entirely fit [73]. The succeeding subsections describe the ML techniques utilized in this investigation. In addition, all models underwent k-fold, statistical, and error assessments, including mean absolute error (MAE), mean absolute percentage error (MAPE), and root mean square error (RMSE). Also, a SHAP analysis is utilized to investigate the effect of input characteristics on the CS of FRNSC. Figure 2 depicts the plan for the study.

#### 2.2.1. Gradient Boosting

**Figure 3:**
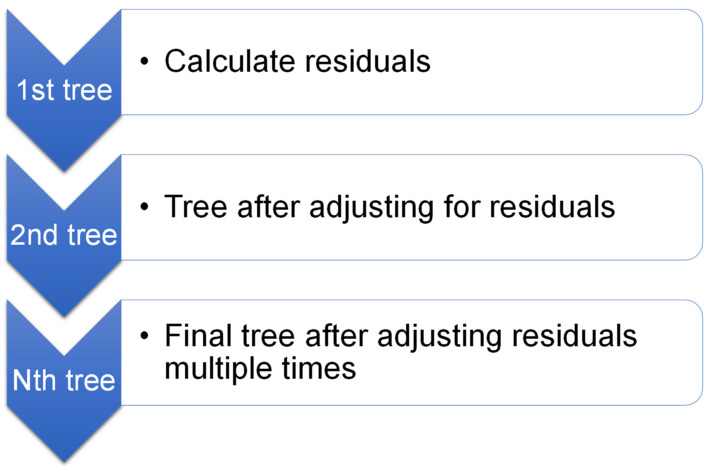
Graphical interpretation of gradient boosting model [76].

In 1999, Friedman [74] suggested GB as an ensemble method for regression and classification. GB is solely beneficial for regression. Figure 3 demonstrates that the GB method associates each repeat of the randomly chosen training dataset with the fundamental model. By randomly subsampling the training dataset, which also inhibits overfitting, it is possible to reduce execution time and improve accuracy. Since every repetition of the model must incorporate minimum data, the smaller the training dataset, the quicker the regression. GB approach needs modification parameters, including shrinkage rate and n-trees, where n-trees are the tree numbers to be produced; n-trees should not be retained too little, and the shrinkage aspect, also recognized as the learning rate, must not be kept too high [75].

#### 2.2.2. Random Forest

**Figure 4:**
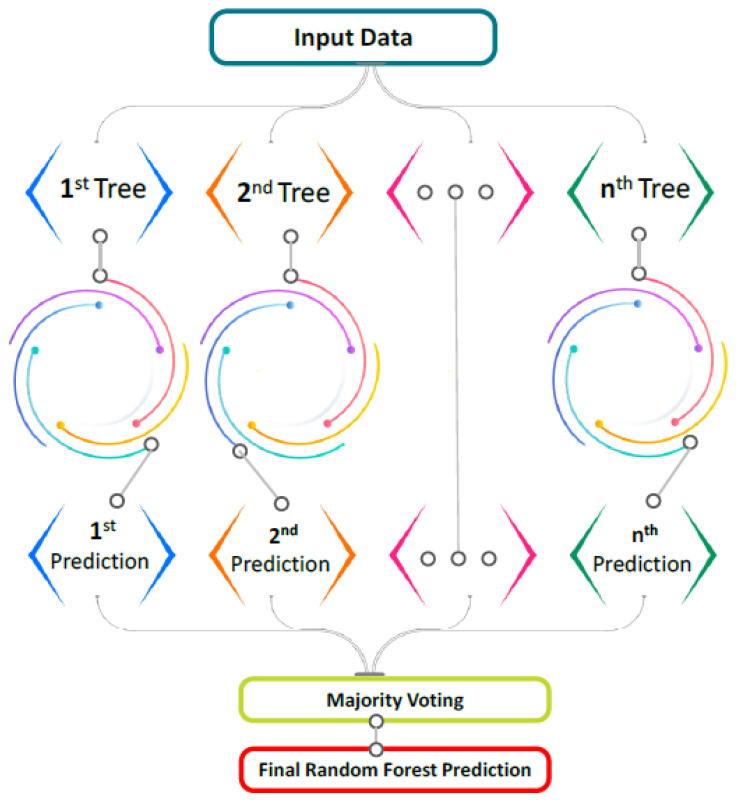
Schematic interpretation of random forest model [80].

The RF method, which is a classification and regression-based technique, is being used frequently [77,78]. In RF, numerous trees, also known as a forest, are constructed, and dissimilar data are arbitrarily picked and assigned to corresponding trees, as seen in Figure 4. Every tree has columns and rows of data, and various measurements of columns and rows are picked. The subsequent processes are performed for the expansion of every tree; two-thirds of the overall data is arbitrarily picked for every tree’s data frame. This practice is considered bagging. The prediction variables are selected at random, and the node separation is accomplished by finely dividing these variables. The leftover data are used to approximate out-of-bag error for all trees. Therefore, the ultimate out-of-bag error rate is determined by merging the mistakes from every tree. Every tree gives regression, and the forest with the greatest number of votes is chosen as the model. The importance of a vote can be either a 1 or a 0. The probability of prediction is measured by the fraction of ones obtained. RF is the most complex method for ensemble learning. It has ideal characteristics for variable importance measures (VIMs), including fewer model parameters and robust overfitting resilience. A decision tree is utilized as a basic forecaster for RF, and RF models with default parameter settings can give acceptable results [79].

#### 2.2.3. Bagging Regressor

**Figure 5:**
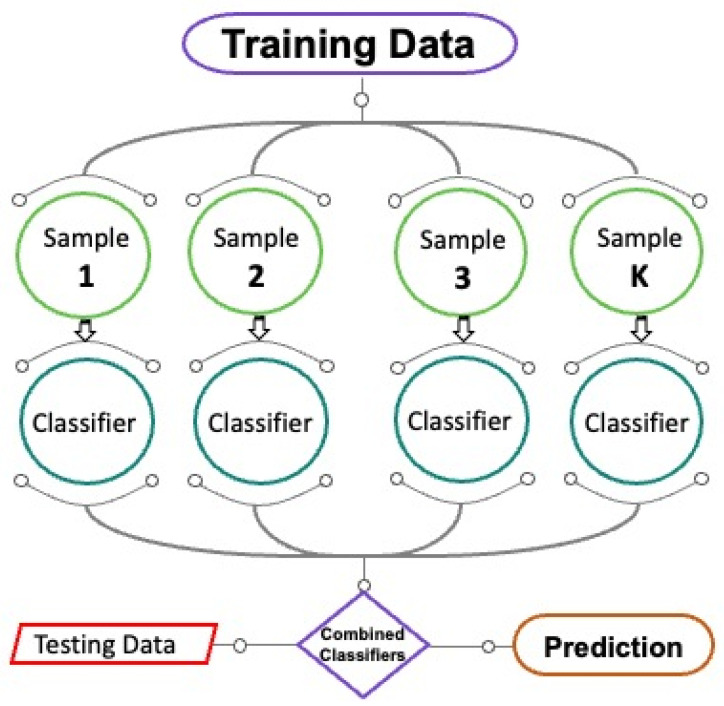
Schematic interpretation of bagging regressor model [80].

Figure 5 depicts a schematic flowchart of the method for BR. It is essentially an analogous ensemble approach that characterizes the forecast model adjustment with the addition of more training data. The irregular sampling method comprises the replacement of data from the main set. Utilizing replacement sampling, every new training data set is able to replicate certain observations. In the bagging process, each component has an equal chance of occurring in the new dataset. The size of the training set is independent of predictive force. In addition, variation may be significantly reduced by fine-tuning the intended outcome estimate. Using these data sets, more models are trained. The mean of all model forecasts is utilized for this ensemble. In regression, the mean of the forecasts of many models can serve as a forecast [81]. Twenty sub-models are used to fine-tune the bagging method using a decision tree in order to determine the optimal output-producing value.

#### 2.2.4. AdaBoost Regressor

**Figure 6:**
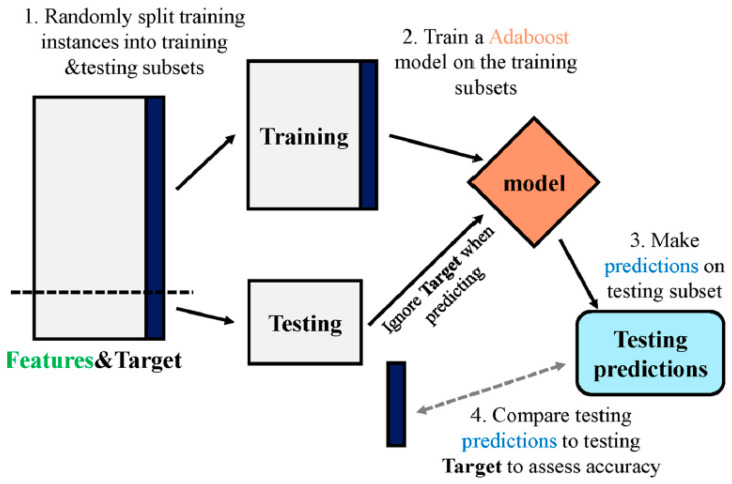
Schematic interpretation of AdaBoost regressor model [83].

Figure 6 depicts the procedure for predicting the outcome of the AR algorithm. As multi-classifiers, multiple algorithms are pooled to form an ensemble, a group of about a thousand learners working toward the same purpose to resolve the situation. An AR method employs ensemble learning, which is essentially a supervised ML method. It is also known as adaptive boosting due to the fact that weights are re-connected to each occurrence, with bigger weights being linked to examples that have been inaccurately grouped. Boosting strategies are usually used to decrease variance and bias in supervised ML. Using ensemble strategies can help weak learners improve. It employs an unlimited amount of decision trees for input data throughout the training stage. During the creation of the first decision tree, incorrectly classified data are highlighted inside the primary model. The identical data records serve as input for a separate model. The above-mentioned procedure would be continued until a specific number of base learners were produced. AR enhances the development of the decision tree’s operation on binary classification problems. Moreover, it is utilized to enhance the ML model’s performance. It is very helpful when employed with weak learners. These ensemble methods are widely employed in material science, specifically for forecasting the mechanical characteristics of cementitious materials [82].

## 3. Analysis of Results

### 3.1. Gradient Boosting Model

**Figure 7:**
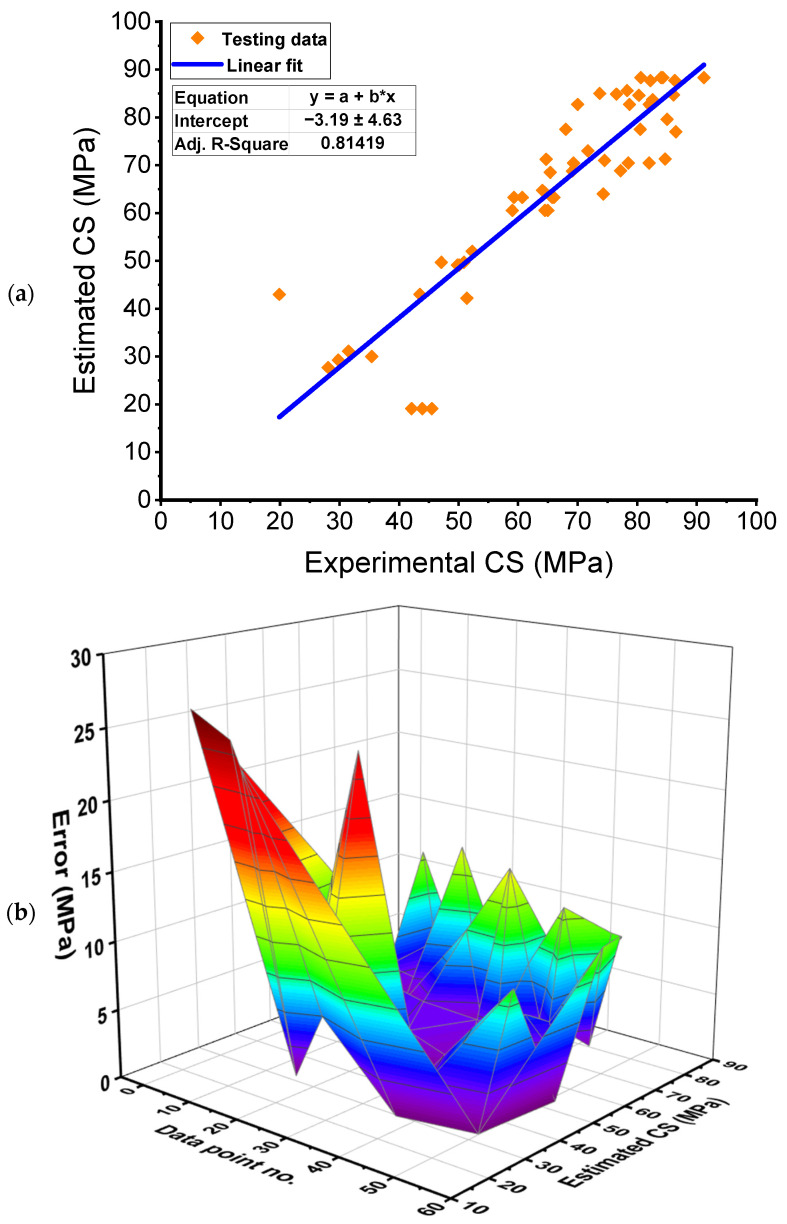
Gradient boosting model: (**a**) Relation between real and anticipated outcomes; (**b**) Dispersion of anticipated results and errors. Error=|Experimental result−Estimated result|.

Figure 7 displays the outcomes of the GB technique for the CS estimation of the FRNSC. Figure 7a depicts the relation among actual data and predicted results. The GB approach produced outcomes with a reasonable level of exactness and a little difference among actual and estimated results. The R^2^ of 0.81 indicates that the GB approach is reasonable in predicting the CS of FRNSC. The dispersal of projected and divergent values (errors) for the GB model is shown in Figure 7b. After examining the error values, the lowest, average, and maximum values were determined to be 0.30 MPa, 5.92 MPa, and 26.40 MPa, respectively. Furthermore, the proportional dispersal of errors was analyzed, and it was found that 16.98% of the values fell below 1 MPa, 24.53% fell within the range of 1–3 MPa, 24.53% fell within the range of 3–6 MPa, 16.98% fell within the range of 6–10 MPa, and 16.98% were greater than 10 MPa. Furthermore, the divergent values show that the GB approach predicted the CS of FRNSC with a satisfactory degree of accuracy.

### 3.2. Random Forest Model

**Figure 8:**
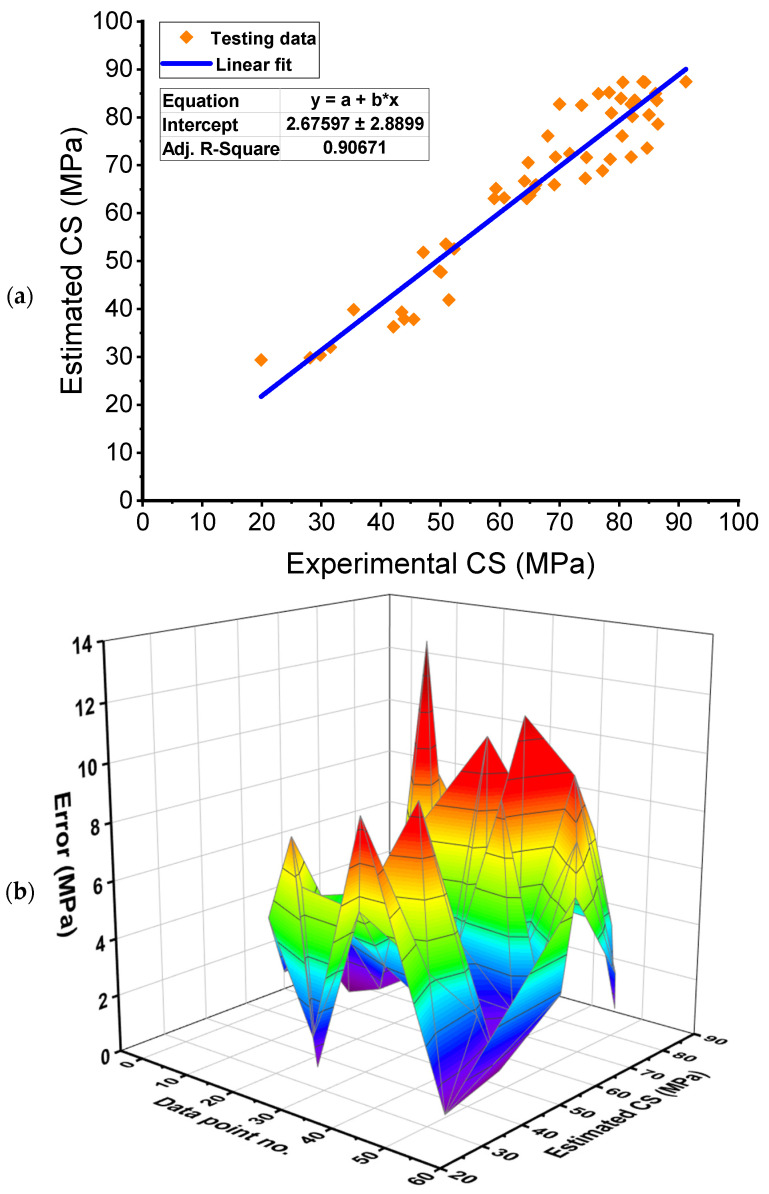
Random forest model: (**a**) Relation between real and anticipated outcomes; (**b**) Dispersion of anticipated results and errors. Error=|Experimental result−Estimated result|.

Figure 8 provide an assessment of the experimental and predicted results of the RF model. Figure 8a shows the relation among real and predicted findings, with an R^2^ of 0.91 demonstrating that the RF approach is more exact than the GB in calculating the CS of FRNSC. Figure 8b represents the spreading of anticipated results and errors using the RF method. The lowest, average, and greatest errors were found to be 0.06 MPa, 4.38 MPa, and 12.77 MPa, respectively. Also, it was determined that 15.09% of the error distribution were below 1 MPa, 28.30% fell between 1–3 MPa, 26.42% fell between 3–6 MPa, 24.53% fell between 6–10 MPa, and 5.66% exceeded 10 MPa. Moreover, this reduction in error suggests that the RF model is more exact than the GB model. The enhanced precision of the RF is a result of its optimal properties for VIMs.

### 3.3. Bagging Regressor Model

**Figure 9:**
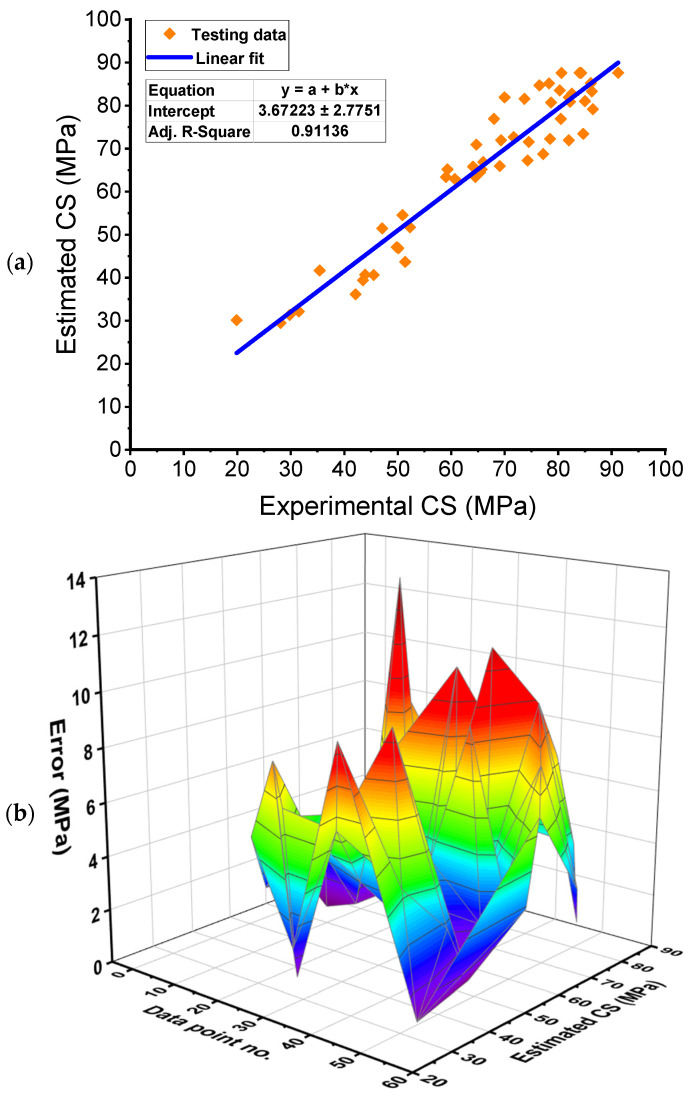
Bagging regressor model: (**a**) Relation between real and anticipated outcomes; (**b**) Dispersion of anticipated results and errors. Error=|Experimental result−Estimated result|.

Figure 9 illustrates the results of the BR technique used to forecast the FRNSC’s CS. Figure 9a shows the link among experimental and projected results. The BR technique produced outcomes with greater precision than the GB model and a lower variance among actual and anticipated findings. With an R^2^ of 0.91, the BR model is equivalent to the RF model in calculating the CS of FRNSC. Figure 9b represents the dispersion of anticipated values and errors using the BR technique. It was revealed that the least, average, and highest error values were 0.10 MPa, 4.24 MPa, and 11.90 MPa, respectively. The error division was 18.87% less than 1 MPa, 18.87% among 1–3 MPa, 32.08% between 3–6 MPa, 22.64% between 6–10 MPa, and 7.55% over 10 MPa. The error distribution also demonstrated that the BR model was more exact than the GB model and had precision equivalent to the RF model.

### 3.4. AdaBoost Regressor Model

**Figure 10:**
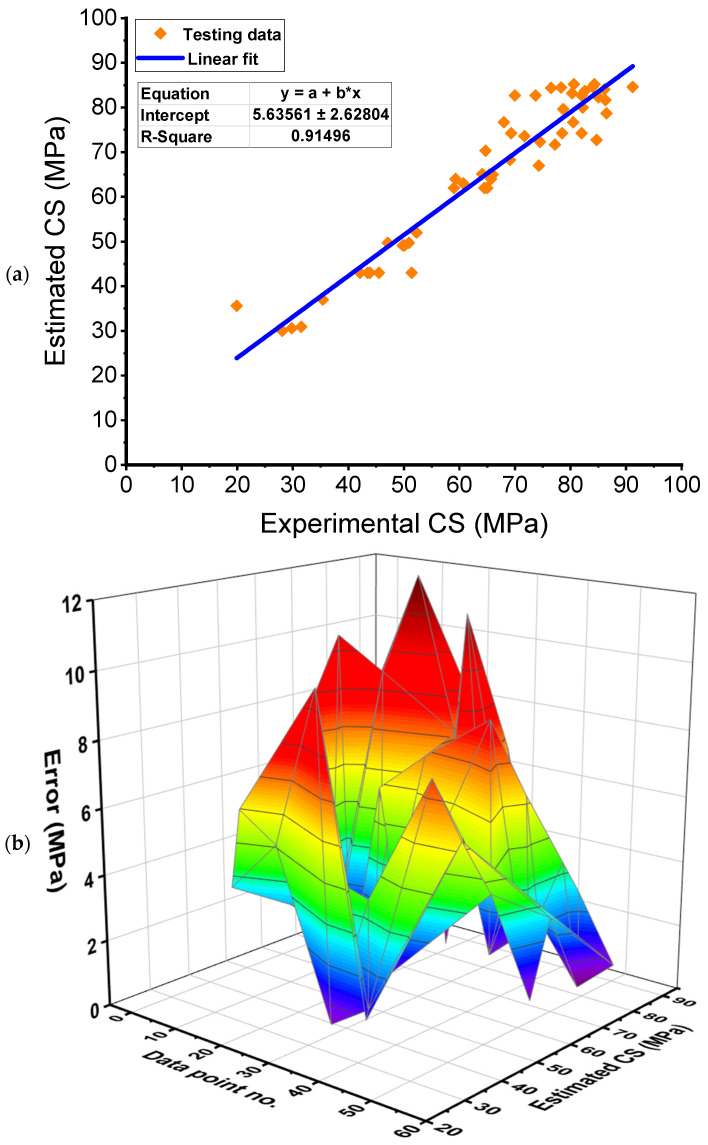
AdaBoost regressor model: (**a**) Relation between real and anticipated outcomes; (**b**) Dispersion of anticipated results and errors. Error=|Experimental result−Estimated result|.

Figure 10 illustrates the results of the AR technique used to forecast the FRNSC’s CS. Figure 10a shows the correlation among actual and projected outcomes. The AR technique produced outcomes with the best precision of all the other models employed in the present study and the least variance among actual and forecasted findings. With an R^2^ of 0.92, the AR model is most exact in calculating the CS of FRNSC. Figure 10b represents the spreading of expected findings and errors using the AR technique. It was found that the least, average, and highest error values were 0.30 MPa, 3.73 MPa, and 15.70 MPa, respectively. The error division was 24.53% less than 1 MPa, 33.96% between 1–3 MPa, 18.87% between 3–6 MPa, 16.98% between 6–10 MPa, and 5.66% over 10 MPa. The error distribution also demonstrated that the AR model is the most exact than the other model employed. The reasons for the best accuracy of the AR model are because it uses an endless amount of decision trees for training, and in the first decision tree, incorrectly categorized data are prioritized. Also, another model uses the same data records. The aforementioned technique is repeated until enough basic learners are created. In addition, AR improves decision tree performance in binary classification.

## 4. Validation

**Figure 11:**
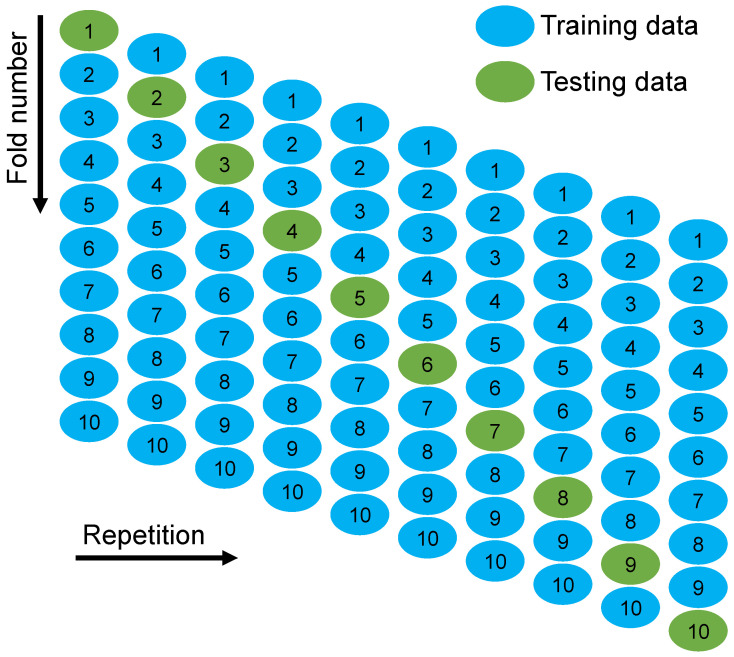
Schematic illustration of the k-fold method [87].

Statistical checks, as well as k-fold methods, were applied to verify the exactness of ML algorithms in use. Typically, statistical checks in the form of errors (MAE, MAPE, and RMSE) are calculated to measure and compare the performance of ML techniques. Also, the k-fold method is applied to test the soundness of an approach by randomly distributing and splitting relevant data into 10 groups [84]. As shown in Figure 11, nine groups are used to train ML models, while one is used to validate them. The ML approach is more accurate when the errors (MAE, MAPE, and RMSE) are minor and R^2^ is larger. In addition, the technique must be performed 10 times for a desirable outcome. This repetitive effort adds substantially to the ML model’s excellent exactness. Also, as shown in Table 2, each model’s accuracy was statistically evaluated using errors assessment (MAE, MEPE, and RMSE). Using Equations (1)–(3) derived from previous research [85,86], the projecting performance of the ML methods was statistically evaluated. It was found that the MAE values for GB, RF, BR, and AR are 5.920, 4.379, 4.237 and 3.727 MPa, respectively. MAPE for GB, RF, BR, and AR were determined to be 11.2%, 7.40%, 7.30%, and 6.50%, respectively. Also, RMSE values for GB, RF, BR, and AR were calculated to be 8.685, 5.416, 5.241 and 5.099 MPa, respectively. These assessments also indicated that the AR model is more precise than the alternatives due to its lower error rate.

(1) $$\mathrm{MAE}=\frac{1}{n}\sum_{i=1}^{n}\left|P_{i}-Ti\right|,$$

(2) $$\mathrm{RMSE}=\sqrt{\sum \frac{{\left(P_{i}-T_{i}\right)}^{2}}{n}},$$

(3) $$\mathrm{MAPE}=\frac{100\%}{n}\sum_{i=1}^{n}\frac{\left|P_{i}-Ti\right|}{T_{i}},$$

where n = size of the dataset, $P_{i}\;$= estimated results, and $T_{i}$ = experimental results.

**Figure 12:**
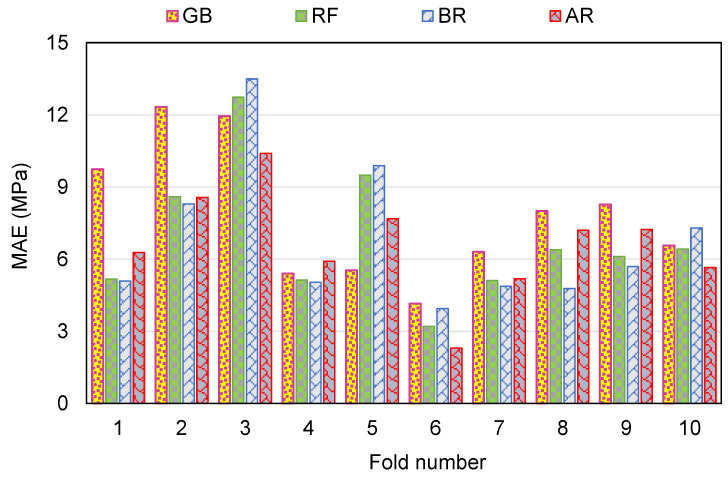
MAE dispersal from k-fold evaluation.

**Figure 13:**
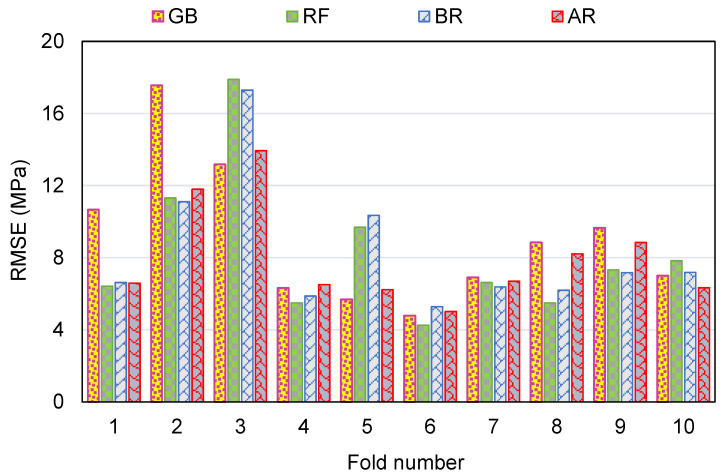
RMSE dispersal from k-fold evaluation.

**Figure 14:**
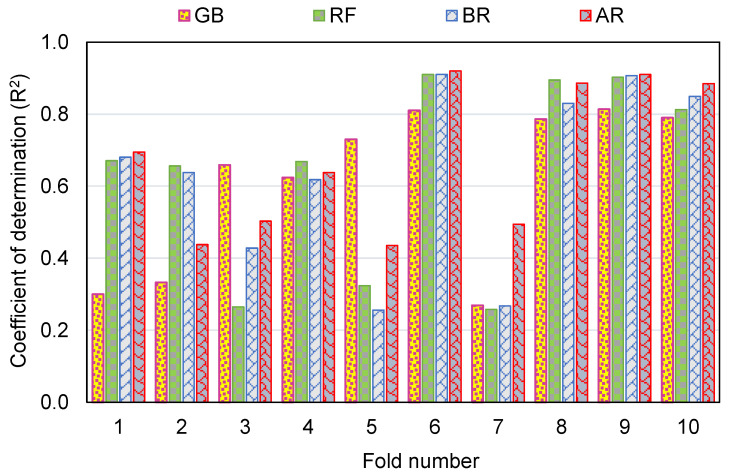
R^2^ dispersal from k-fold evaluation.

To measure the validity of models using k-fold evaluation, R^2^, RMSE, and MAE were calculated, and their results are shown in Table 3. To assess the outputs of each ML method’s k-fold analysis, Figure 12, Figure 13 and Figure 14 are created. The MAE values for the GB method ranged from 4.16 to 12.33 MPa, with a mean of 7.83 MPa. The range of MAE for the RF model was 3.21 to 12.73 MPa, with a mean of 6.84 MPa. Also, the MAE values for the BR method varied among 3.94 and 13.50 MPa, with a mean of 6.84 MPa. In addition, the MAE values for the AR method varied among 2.30 and 10.40 MPa, with a mean of 6.64 MPa (Figure 12). Similarly, average RMSE values for the GB, RF, BR, and AR models were 9.06, 8.23, 8.34 and 8.01 MPa, respectively (Figure 13). Though the average R^2^ values for GB, RF, BR, and AR were 0.61, 0.64, 0.64 and 0.68, respectively (Figure 14). In terms of forecasting the CS of FRNSC, the AR model with the lowest error values and the highest R^2^ is comparably the most accurate.

## 5. Interaction and Impact of Input Features on the CS of FRNSC

**Figure 15:**
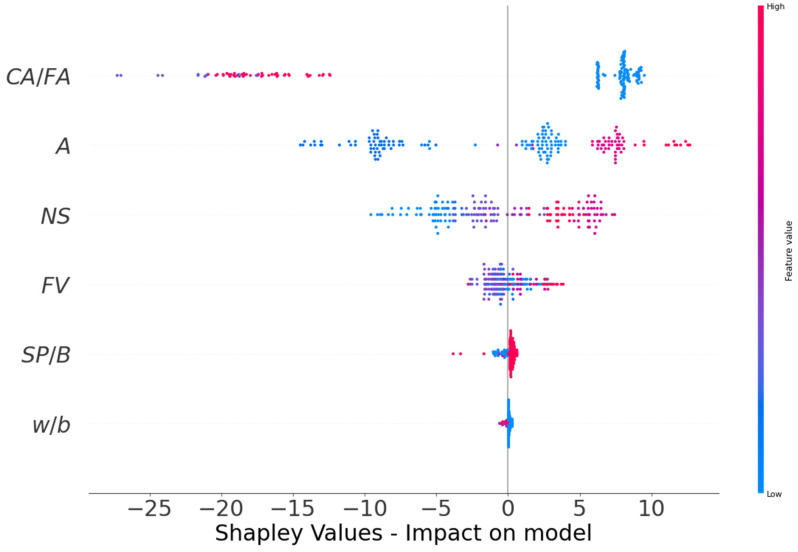
SHAP graph illustrating the impact of input features on ML models.

In this study, the effect of input features on the performance of the CS of FRNSC was examined. SHAP tree explainer is primarily applied to the entire dataset to deliver a more precise account of global feature effects by integrating local SHAP explanations. Figure 15 depicts the findings of the violin SHAP plot for all of the input parameters utilized in this study. In this graph, each parameter value is denoted by a distinctive color, and the matching SHAP value on the x-axis represents the influence of an input feature. CA/FA is an example of an input characteristic with a greater effect, illustrative of the stronger negative correlation among this feature and the CS of FRNSC (higher red spots on the negative axis). This suggests that a rise in CA/FA would likely result in a decrease in CS. However, the age of specimen (A) has a more positive impact (more red dots on the positive side), suggesting that at increased specimen age, CS improves. The impact of NS and FV on the CS was determined to be both positive and negative, implying that the incorporation of NS and FV up to an optimal amount has a positive impact while using NS and FV above that limit has a negative impact on the CS of FRNSC. A similar correlation of SP/B on CS to that of NS can also be seen. The impact of the w/b was determined to be unclear due to the less variation of w/b in the used dataset. Employing a greater size dataset with a higher variation of input features might produce better relationships.

**Figure 16:**
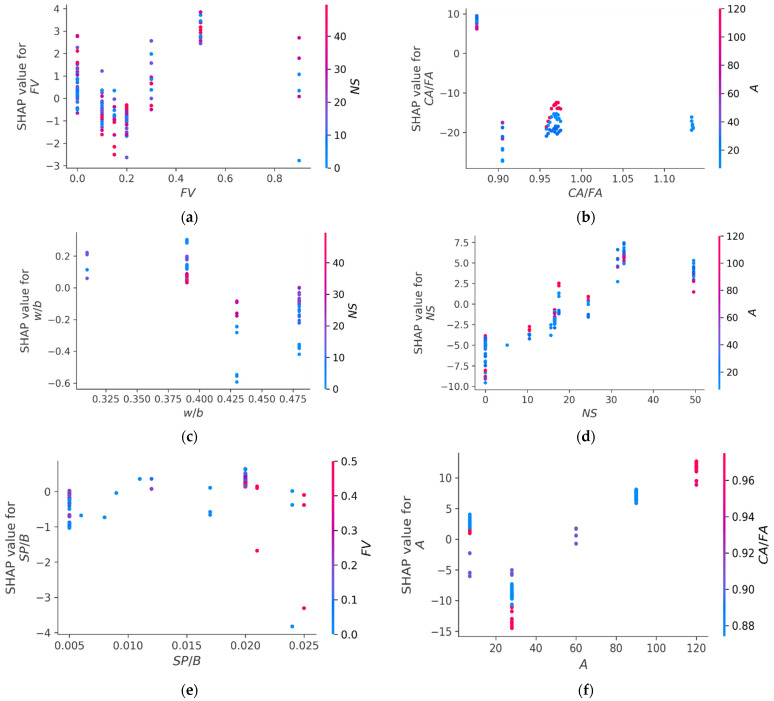
Interaction graphs: (**a**) Fiber volume; (**b**) Coarse aggregate to fine aggregate ratio; (**c**) water to binder ratio; (**d**) Nano-silica; (**e**) Superplasticizer to binder ratio (**f**) Age of specimen.

Figure 16 demonstrates the relationships between the input parameters and their impact on the CS of the FRNSC. Figure 16a displays the FV interaction. The scatter figure demonstrates that, amongst other features, FV has the greatest influence on the CS of FRNSC, which increases with the quantity of FV up to 0.5% and then decreases and interacts mostly with the NS. Under these conditions, an FV of nearly 0.5% is optimal for achieving a high CS for FRNSC while using the same components as in the current study. Conversely, increasing levels of CA/FA have a negative influence on the CS of FRNSC (Figure 16b) and interact mostly with the age of the specimen. Also, as depicted in Figure 16c, w/b interacts mostly with NS and increasing its value has a negative impact on the CS of FRNSC. Thus, the w/b should be maintained lower to achieve higher strength. The impact of incorporating NS in concrete was found to be beneficial (see Figure 16d). Using NS up to an optimal quantity will help improve the strength of concrete. Thus, NS might be used in the range of 30–35 kg/m^3^ to obtain enhanced material strength. In addition, NS interacts mostly with the age of specimen (A), among the other input features. This suggests that the development of concrete strength with NS is proportional to the specimen age, i.e., at increased age, the strength will increase. As seen from Figure 16e, the higher SP/B ratio deteriorated the FRNSC strength, and its optimal ratio is nearly 0.20. Figure 16f implies that with increasing specimen age, the CS of FRNSC increases and maximum strength might be achieved at 120 days. It is essential to note that these findings are based on the kinds of input features and amount of data samples analyzed in this research. Employing diverse input features and data samples may result in unique outcomes.

## 6. Discussion

This study employed four ensemble ML methods, including GB, RF, BR, and AR. The accuracy of each method was assessed to find out which is the highly efficient predictor. Compared to the GB method, with an R^2^ of 0.81, the other three models, i.e., RF, BR, and AR, produced more accurate results with an R^2^ of 0.91, 0.91, and 0.92, respectively. The accuracy of the RF, BR, and RF was found to be approximately comparable in predicting the CS of FRNSC from the R^2^ and error distributions (MAE, MAPE, and RMSE). For the comparison of the results of the present study to those of the previously published literature, Table 4 has been constructed. The past studies also reported the higher precision of the RF, BR, and AR models in forecasting the strength characteristics of concretes [67,76,79,87,88]. For example, Khan et al. [67] employed two ensemble ML methods (GB and BR) to anticipate the CS of recycled aggregate concrete and found the best accurate results with the BR model. 

**Figure 17:**
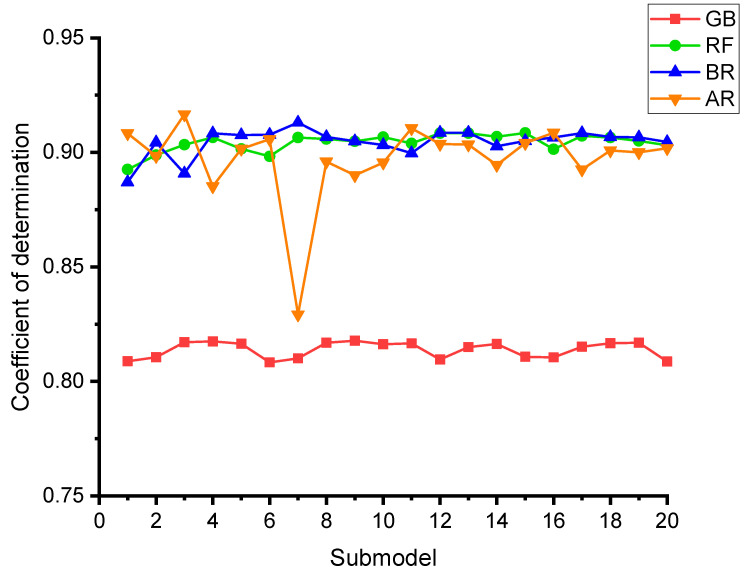
Dispersal of R^2^ for the employed ML models.

In addition, each model’s accuracy was evaluated using statistical and k-fold approaches. A model is more precise when the degree of divergence (errors) from the experimental results is less. Nevertheless, determining and suggesting the most favorable ML approach for predicting properties in diverse research fields is difficult since the precision of an ML technique is largely reliant on the number of inputs and data samples utilized to run algorithms [85]. Ensemble ML approaches commonly use the weak learner by creating sub-models that are trained on the dataset and tuned to increase the R^2^ value, thus yielding outcomes with higher accuracy than the individual ML models. The distribution of R^2^ for the GB, RF, BR, and AR sub-models is seen in Figure 17. The R^2^ values for GB sub-models ranged from 0.808 to 0.818, yielding an average of 0.814. Also, the R^2^ values for the RF sub-models ranged from 0.893 to 0.909, yielding an average of 0.904. Similarly, the average R^2^ of BR and AR sub-models was determined to be 0.905 and 0.917, respectively. These findings validate that the RF, BR, and AR sub-models have higher accuracy than the GB sub-models, having nearly equal precision. In addition, SHAP analysis is carried out utilized to explore the interaction and effect of input features on the CS of FRNSC. CA/FA was shown to be a highly effective input feature, demonstrating a larger negative correlation with FRNSC’s CS. However, the impact of specimen age was found to be more beneficial on the CS of FRNSC. The influence of NS and FV on CS was both positive and negative, signifying that utilizing NS and FV up to an optimum level has a good impact, while using NS and FV over that limit has a detrimental impact on FRNSC’s CS. SP/B also has a comparable correlation. However, due to little variance in w/b in the data sample, the w/b influence b’s was unclear, and larger datasets with more input attributes may create better relationships. This sort of exploration will support the building sector by accelerating the progress of quick and economical approaches for calculating material properties and the impact of raw ingredients.

## 7. Conclusions

This study focused on comparing the performance of ensemble machine learning (ML) techniques to predict the compressive strength (CS) of fiber-reinforced nano-silica concrete (FRNSC). Four types of ML methods, including gradient boosting (GB), random forest (RF), bagging regressor (BR), and AdaBoost regressor (AR), were used to forecast outcomes. In addition, SHAP analysis was performed to assess the interaction of input features and their impact on the CS of FRNSC. This research reached the following conclusions:

- The performance of the GB model in estimating the CS of FRNSC was found to be satisfactory, with an R^2^ of 0.81, while the performance of other ML models, i.e., RF, BR, and AR, was found to be more accurate in anticipating the CS of FRNSC, with an R^2^ of 0.91, 0.91, and 0.92, respectively. The accuracy of RF, BR, and AR might be considered approximately equal.
- The difference between estimated and experimental results (errors) for all models was analyzed and revealed that in 16.98%, 5.66%, 7.55%, and 5.66% of estimated results, the error values for GB, RF, BR, and AR models were above 10 MPa, respectively. These errors also confirmed the comparable precision of RF, BR, and AR models and higher accuracy than the GB model.
- Statistical and k-fold assessments were employed to confirm the employed model’s performance. Smaller errors and higher R^2^ reflect ML model accuracy. The mean absolute percentage error (MAPE) for the GB, RF, BR, and AR models was 11.2%, 7.40%, 7.30%, and 6.50%. These MAPEs further validated the best performance of the AR model, followed by BR, RF, and GB in predicting the CS of FRNSC.
- K-fold analysis revealed that the average MAE for GB, RF, BR, and AR was 7.83, 6.84, 6.84, and 6.64 MPa, respectively. Similarly, the average RMSE for GB, RF, BR, and AR was 9.06, 8.23, 8.34, and 8.01, respectively. In contrast, the average R^2^ for GB, RF, BR, and AR was 0.61, 0.64, 0.64, and 0.68, respectively. The lower errors (MAE and RMSE) and higher R^2^ suggested the AR model had the highest precision among the others.
- SHAP analysis revealed that coarse aggregate to fine aggregate ratio (CA/FA) had a stronger adverse correlation with FRNSC’s CS. Whereas specimen age had a positive impact on FRNSC CS and nano-silica (NS), fiber volume (FV), and superplasticizer to binder ratio (SP/B) had both favorable and detrimental effects on the CS of FRNSC. Using NS, FV, and SP/B within the optimum limits enhances the CS, while their usage in lower and higher concentrations may cause deterioration of CS.
- This sort of exploration will support the building sector by accelerating the progress of quick and economical approaches for calculating material properties and the impact of raw ingredients.

## Figures and Tables

**Table 1:** The outcomes of the descriptive assessment of input parameters.

Parameter	FV (%)	CA/FA	w/b	NS (kg/m^3^)	SP/B	A (Days)
Mean	0.20	0.91	0.41	21.21	0.02	41.65
Median	0.20	0.87	0.39	16.50	0.02	28.00
Mode	0.20	0.87	0.39	0.00	0.02	7.00
Standard Deviation	0.18	0.06	0.04	17.30	0.01	38.25
Range	0.90	0.26	0.17	49.60	0.02	113.00
Minimum	0.00	0.87	0.31	0.00	0.01	7.00
Maximum	0.90	1.14	0.48	49.60	0.03	120.00

**Table 2:** Statistical evaluation of the techniques used.

Model	MAE (MPa)	MAPE (%)	RMSE (MPa)
Gradient boosting	5.920	11.2	8.685
Random forest	4.379	7.40	5.416
Bagging regressor	4.237	7.30	5.241
AdaBoost regressor	3.727	6.50	5.099

**Table 3:** MAE, RMSE, and R^2^ results from the k-fold assessment.

K-Fold	GB			RF			BR			AR		
	MAE	RMSE	R^2^	MAE	RMSE	R^2^	MAE	RMSE	R^2^	MAE	RMSE	R^2^
1	9.73	10.66	0.30	5.17	6.42	0.67	5.10	6.62	0.68	6.27	6.59	0.69
2	12.33	17.56	0.33	8.60	11.32	0.66	8.30	11.10	0.64	8.56	11.79	0.44
3	11.94	13.17	0.66	12.73	17.88	0.26	13.50	17.29	0.43	10.40	13.93	0.50
4	5.40	6.32	0.62	5.14	5.49	0.67	5.04	5.86	0.62	5.91	6.50	0.64
5	5.54	5.68	0.73	9.50	9.69	0.32	9.89	10.35	0.26	7.68	6.23	0.44
6	4.16	4.79	0.81	3.21	4.26	0.91	3.94	5.28	0.91	2.30	5.02	0.92
7	6.31	6.91	0.27	5.11	6.62	0.26	4.88	6.37	0.27	5.18	6.68	0.49
8	8.01	8.84	0.79	6.39	5.48	0.90	4.78	6.19	0.83	7.20	8.21	0.89
9	8.28	9.65	0.81	6.10	7.32	0.90	5.69	7.17	0.91	7.24	8.85	0.91
10	6.57	7.00	0.79	6.43	7.82	0.81	7.30	7.18	0.85	5.66	6.33	0.88

**Table 4:** Ensemble machine learning models employed previously.

Type of Material	Forecasted Properties	Ensemble ML Methods Used	No. of Input Features	Size of Dataset	Optional ML Method	Reference
Recycled aggregate concrete	CS	GB and BR	8	638	BR	[67]
Geopolymer concrete	CS	BR and RF	9	371	BR	[88]
High-performance concrete	CS	AR, BR, extreme GB, and RF	8	1030	RF and BR	[85]
Recycled aggregate concrete	CS and flexural strength	GB and RF	12	638	RF	[76]
Geopolymer concrete	CS	AR and RF	9	363	AR and RF	[87]
Geopolymer concrete	CS	BR and AR	9	154	BR	[73]
High-performance concrete	CS	AR and RF	7	1030	RF	[79]
High-performance concrete	CS	AR and BR	8	1030	BR	[89]

## Data Availability

The data used in this research has been properly cited and reported in the main text.

## Supplementary Materials

The following supporting information can be downloaded at: https://www.mdpi.com/article/10.3390/polym14183906/s1, Table S1: Data used for modeling [90,91,92,93].
