# Supplementary material for: Application of Ensemble Machine Learning Methods to Estimate the Compressive Strength of Fiber-Reinforced Nano-Silica Modified Concrete
Source: Polymers (Basel). 2022 Sep 19;14(18):3906. doi: 10.3390/polym14183906 (PMC9506242; doi:10.3390/polym14183906)
Supplement: Supplementary file 1 [file polymers-14-03906-s001.zip › polymers-1882401-supplementary data.pdf]

Table S1: Data used for modeling

| Ref. | Fiber volume (%) | CA/FA | Limestone powder (Kg/m³) | w/b | Nano-silica (Kg/m³) | SP/B | Age (days) | CS (MPa) |
|---|---|---|---|---|---|---|---|---|
| [1] | 0 | 0.874 | 288.9 | 0.39 | 0 | 0.02 | 7 | 73 |
| | 0.2 | 0.874 | 288.9 | 0.39 | 0 | 0.02 | 7 | 74.3 |
| | 0.3 | 0.874 | 288.9 | 0.39 | 0 | 0.02 | 7 | 81.5 |
| | 0.5 | 0.874 | 288.9 | 0.39 | 0 | 0.02 | 7 | 78 |
| | 0.1 | 0.874 | 288.9 | 0.39 | 0 | 0.02 | 7 | 71.7 |
| | 0.15 | 0.874 | 288.9 | 0.39 | 0 | 0.02 | 7 | 69.3 |
| | 0.2 | 0.874 | 288.9 | 0.39 | 0 | 0.02 | 7 | 66.6 |
| | 0.15 | 0.874 | 288.9 | 0.39 | 0 | 0.02 | 7 | 82 |
| | 0.2 | 0.874 | 288.9 | 0.39 | 0 | 0.02 | 7 | 78.5 |
| | 0.3 | 0.874 | 288.9 | 0.39 | 0 | 0.02 | 7 | 72.8 |
| | 0 | 0.874 | 288.9 | 0.39 | 16.5 | 0.02 | 7 | 75 |
| | 0.2 | 0.874 | 288.9 | 0.39 | 16.5 | 0.02 | 7 | 77.5 |
| | 0.3 | 0.874 | 288.9 | 0.39 | 16.5 | 0.02 | 7 | 82.7 |
| | 0.5 | 0.874 | 288.9 | 0.39 | 16.5 | 0.02 | 7 | 78.2 |
| | 0.1 | 0.874 | 288.9 | 0.39 | 16.5 | 0.02 | 7 | 73.6 |
| | 0.15 | 0.874 | 288.9 | 0.39 | 16.5 | 0.02 | 7 | 70.8 |
| | 0.2 | 0.874 | 288.9 | 0.39 | 16.5 | 0.02 | 7 | 68 |
| | 0.15 | 0.874 | 288.9 | 0.39 | 16.5 | 0.02 | 7 | 82.6 |
| | 0.2 | 0.874 | 288.9 | 0.39 | 16.5 | 0.02 | 7 | 80.5 |
| | 0.3 | 0.874 | 288.9 | 0.39 | 16.5 | 0.02 | 7 | 78.7 |
| | 0 | 0.874 | 288.9 | 0.39 | 33 | 0.02 | 7 | 86 |
| | 0.2 | 0.874 | 288.9 | 0.39 | 33 | 0.02 | 7 | 85.7 |
| | 0.3 | 0.874 | 288.9 | 0.39 | 33 | 0.02 | 7 | 88 |
| | 0.5 | 0.874 | 288.9 | 0.39 | 33 | 0.02 | 7 | 87.2 |
| | 0.1 | 0.874 | 288.9 | 0.39 | 33 | 0.02 | 7 | 82.6 |
| | 0.15 | 0.874 | 288.9 | 0.39 | 33 | 0.02 | 7 | 78.7 |
| | 0.2 | 0.874 | 288.9 | 0.39 | 33 | 0.02 | 7 | 76.5 |
| | 0.15 | 0.874 | 288.9 | 0.39 | 33 | 0.02 | 7 | 88.7 |
| | 0.2 | 0.874 | 288.9 | 0.39 | 33 | 0.02 | 7 | 84 |
| | 0.3 | 0.874 | 288.9 | 0.39 | 33 | 0.02 | 7 | 83.6 |
| | 0 | 0.874 | 288.9 | 0.39 | 49.6 | 0.02 | 7 | 85 |
| | 0.2 | 0.874 | 288.9 | 0.39 | 49.6 | 0.02 | 7 | 85.2 |
| | 0.3 | 0.874 | 288.9 | 0.39 | 49.6 | 0.02 | 7 | 86.7 |
| | 0.5 | 0.874 | 288.9 | 0.39 | 49.6 | 0.02 | 7 | 86.1 |
| | 0.1 | 0.874 | 288.9 | 0.39 | 49.6 | 0.02 | 7 | 79.6 |
| | 0.15 | 0.874 | 288.9 | 0.39 | 49.6 | 0.02 | 7 | 77 |
| | 0.2 | 0.874 | 288.9 | 0.39 | 49.6 | 0.02 | 7 | 82.3 |
| | 0.15 | 0.874 | 288.9 | 0.39 | 49.6 | 0.02 | 7 | 86.5 |
| | 0.2 | 0.874 | 288.9 | 0.39 | 49.6 | 0.02 | 7 | 83 |
| | 0.3 | 0.874 | 288.9 | 0.39 | 49.6 | 0.02 | 7 | 82.7 |
| | 0 | 0.958 | 0 | 0.48 | 0 | 0.005 | 7 | 42.1 |
| | 0.1 | 0.96 | 0 | 0.48 | 0 | 0.005 | 7 | 43 |
| | 0 | 0.874 | 288.9 | 0.39 | 0 | 0.02 | 28 | 65 |
| | 0.2 | 0.874 | 288.9 | 0.39 | 0 | 0.02 | 28 | 62 |
| | 0.3 | 0.874 | 288.9 | 0.39 | 0 | 0.02 | 28 | 64.5 |
| | 0.5 | 0.874 | 288.9 | 0.39 | 0 | 0.02 | 28 | 65 |
| | 0.1 | 0.874 | 288.9 | 0.39 | 0 | 0.02 | 28 | 64.5 |
| | 0.15 | 0.874 | 288.9 | 0.39 | 0 | 0.02 | 28 | 63.7 |
| | 0.2 | 0.874 | 288.9 | 0.39 | 0 | 0.02 | 28 | 60.6 |

| | | | | | | | |
|---|---|---|---|---|---|---|---|
| 0.15 | 0.874 | 288.9 | 0.39 | 0 | 0.02 | 28 | 61 |
| 0.2 | 0.874 | 288.9 | 0.39 | 0 | 0.02 | 28 | 59 |
| 0.3 | 0.874 | 288.9 | 0.39 | 0 | 0.02 | 28 | 59 |
| 0 | 0.874 | 288.9 | 0.39 | 16.5 | 0.02 | 28 | 68.5 |
| 0.2 | 0.874 | 288.9 | 0.39 | 16.5 | 0.02 | 28 | 65 |
| 0.3 | 0.874 | 288.9 | 0.39 | 16.5 | 0.02 | 28 | 65.7 |
| 0.5 | 0.874 | 288.9 | 0.39 | 16.5 | 0.02 | 28 | 66 |
| 0.1 | 0.874 | 288.9 | 0.39 | 16.5 | 0.02 | 28 | 65.4 |
| 0.15 | 0.874 | 288.9 | 0.39 | 16.5 | 0.02 | 28 | 64 |
| 0.2 | 0.874 | 288.9 | 0.39 | 16.5 | 0.02 | 28 | 61.5 |
| 0.15 | 0.874 | 288.9 | 0.39 | 16.5 | 0.02 | 28 | 62.5 |
| 0.2 | 0.874 | 288.9 | 0.39 | 16.5 | 0.02 | 28 | 60.7 |
| 0.3 | 0.874 | 288.9 | 0.39 | 16.5 | 0.02 | 28 | 59.3 |
| 0 | 0.874 | 288.9 | 0.39 | 33 | 0.02 | 28 | 78.7 |
| 0.2 | 0.874 | 288.9 | 0.39 | 33 | 0.02 | 28 | 77.2 |
| 0.3 | 0.874 | 288.9 | 0.39 | 33 | 0.02 | 28 | 79.2 |
| 0.5 | 0.874 | 288.9 | 0.39 | 33 | 0.02 | 28 | 79 |
| 0.1 | 0.874 | 288.9 | 0.39 | 33 | 0.02 | 28 | 74.5 |
| 0.15 | 0.874 | 288.9 | 0.39 | 33 | 0.02 | 28 | 72.5 |
| 0.2 | 0.874 | 288.9 | 0.39 | 33 | 0.02 | 28 | 71.7 |
| 0.15 | 0.874 | 288.9 | 0.39 | 33 | 0.02 | 28 | 69.5 |
| 0.2 | 0.874 | 288.9 | 0.39 | 33 | 0.02 | 28 | 66 |
| 0.3 | 0.874 | 288.9 | 0.39 | 33 | 0.02 | 28 | 64.4 |
| 0 | 0.874 | 288.9 | 0.39 | 49.6 | 0.02 | 28 | 75 |
| 0.2 | 0.874 | 288.9 | 0.39 | 49.6 | 0.02 | 28 | 74 |
| 0.3 | 0.874 | 288.9 | 0.39 | 49.6 | 0.02 | 28 | 74.3 |
| 0.5 | 0.874 | 288.9 | 0.39 | 49.6 | 0.02 | 28 | 73.2 |
| 0.1 | 0.874 | 288.9 | 0.39 | 49.6 | 0.02 | 28 | 72.3 |
| 0.15 | 0.874 | 288.9 | 0.39 | 49.6 | 0.02 | 28 | 67.4 |
| 0.2 | 0.874 | 288.9 | 0.39 | 49.6 | 0.02 | 28 | 68.5 |
| 0.15 | 0.874 | 288.9 | 0.39 | 49.6 | 0.02 | 28 | 67 |
| 0.2 | 0.874 | 288.9 | 0.39 | 49.6 | 0.02 | 28 | 64.7 |
| 0.3 | 0.874 | 288.9 | 0.39 | 49.6 | 0.02 | 28 | 64 |
| 0 | 0.874 | 288.9 | 0.39 | 0 | 0.02 | 90 | 80 |
| 0.2 | 0.874 | 288.9 | 0.39 | 0 | 0.02 | 90 | 81 |
| 0.3 | 0.874 | 288.9 | 0.39 | 0 | 0.02 | 90 | 85.3 |
| 0.5 | 0.874 | 288.9 | 0.39 | 0 | 0.02 | 90 | 83 |
| 0.1 | 0.874 | 288.9 | 0.39 | 0 | 0.02 | 90 | 74.8 |
| 0.15 | 0.874 | 288.9 | 0.39 | 0 | 0.02 | 90 | 71.3 |
| 0.2 | 0.874 | 288.9 | 0.39 | 0 | 0.02 | 90 | 68.2 |
| 0.15 | 0.874 | 288.9 | 0.39 | 0 | 0.02 | 90 | 84.7 |
| 0.2 | 0.874 | 288.9 | 0.39 | 0 | 0.02 | 90 | 80 |
| 0.3 | 0.874 | 288.9 | 0.39 | 0 | 0.02 | 90 | 77 |
| 0 | 0.874 | 288.9 | 0.39 | 16.5 | 0.02 | 90 | 82.2 |
| 0.2 | 0.874 | 288.9 | 0.39 | 16.5 | 0.02 | 90 | 82 |
| 0.3 | 0.874 | 288.9 | 0.39 | 16.5 | 0.02 | 90 | 84.6 |
| 0.5 | 0.874 | 288.9 | 0.39 | 16.5 | 0.02 | 90 | 82.5 |
| 0.1 | 0.874 | 288.9 | 0.39 | 16.5 | 0.02 | 90 | 75 |
| 0.15 | 0.874 | 288.9 | 0.39 | 16.5 | 0.02 | 90 | 73.7 |
| 0.2 | 0.874 | 288.9 | 0.39 | 16.5 | 0.02 | 90 | 70 |
| 0.15 | 0.874 | 288.9 | 0.39 | 16.5 | 0.02 | 90 | 85 |
| 0.2 | 0.874 | 288.9 | 0.39 | 16.5 | 0.02 | 90 | 82.7 |
| 0.3 | 0.874 | 288.9 | 0.39 | 16.5 | 0.02 | 90 | 80.3 |

| | | | | | | | | |
|---|---|---|---|---|---|---|---|---|
| | 0 | 0.874 | 288.9 | 0.39 | 33 | 0.02 | 90 | 87.7 |
| | 0.2 | 0.874 | 288.9 | 0.39 | 33 | 0.02 | 90 | 88.3 |
| | 0.3 | 0.874 | 288.9 | 0.39 | 33 | 0.02 | 90 | 91.2 |
| | 0.5 | 0.874 | 288.9 | 0.39 | 33 | 0.02 | 90 | 87.5 |
| | 0.1 | 0.874 | 288.9 | 0.39 | 33 | 0.02 | 90 | 83.2 |
| | 0.15 | 0.874 | 288.9 | 0.39 | 33 | 0.02 | 90 | 81.1 |
| | 0.2 | 0.874 | 288.9 | 0.39 | 33 | 0.02 | 90 | 80.6 |
| | 0.15 | 0.874 | 288.9 | 0.39 | 33 | 0.02 | 90 | 89.8 |
| | 0.2 | 0.874 | 288.9 | 0.39 | 33 | 0.02 | 90 | 84.3 |
| | 0.3 | 0.874 | 288.9 | 0.39 | 33 | 0.02 | 90 | 84 |
| | 0 | 0.874 | 288.9 | 0.39 | 49.6 | 0.02 | 90 | 86.3 |
| | 0.2 | 0.874 | 288.9 | 0.39 | 49.6 | 0.02 | 90 | 86.7 |
| | 0.3 | 0.874 | 288.9 | 0.39 | 49.6 | 0.02 | 90 | 87 |
| | 0.5 | 0.874 | 288.9 | 0.39 | 49.6 | 0.02 | 90 | 86 |
| | 0.1 | 0.874 | 288.9 | 0.39 | 49.6 | 0.02 | 90 | 81.7 |
| | 0.15 | 0.874 | 288.9 | 0.39 | 49.6 | 0.02 | 90 | 82.3 |
| | 0.2 | 0.874 | 288.9 | 0.39 | 49.6 | 0.02 | 90 | 78.3 |
| | 0.15 | 0.874 | 288.9 | 0.39 | 49.6 | 0.02 | 90 | 78.2 |
| | 0.2 | 0.874 | 288.9 | 0.39 | 49.6 | 0.02 | 90 | 84.5 |
| | 0.3 | 0.874 | 288.9 | 0.39 | 49.6 | 0.02 | 90 | 84 |
| [2] | 0.2 | 0.962 | 0 | 0.48 | 0 | 0.005 | 7 | 43.5 |
| | 0 | 0.964 | 0 | 0.48 | 10.5 | 0.005 | 7 | 49.1 |
| | 0.1 | 0.967 | 0 | 0.48 | 10.5 | 0.005 | 7 | 49.8 |
| | 0.2 | 0.969 | 0 | 0.48 | 10.5 | 0.005 | 7 | 50.1 |
| | 0 | 0.968 | 0 | 0.48 | 17.5 | 0.005 | 7 | 54.8 |
| | 0.1 | 0.97 | 0 | 0.48 | 17.5 | 0.005 | 7 | 54.8 |
| | 0.2 | 0.972 | 0 | 0.48 | 17.5 | 0.005 | 7 | 55 |
| | 0 | 0.971 | 0 | 0.48 | 24.5 | 0.005 | 7 | 52 |
| | 0.1 | 0.973 | 0 | 0.48 | 24.5 | 0.005 | 7 | 52.3 |
| | 0.2 | 0.975 | 0 | 0.48 | 24.5 | 0.005 | 7 | 52 |
| | 0 | 0.958 | 0 | 0.48 | 0 | 0.005 | 28 | 27.1 |
| | 0.1 | 0.96 | 0 | 0.48 | 0 | 0.005 | 28 | 27.7 |
| | 0.2 | 0.962 | 0 | 0.48 | 0 | 0.005 | 28 | 28.1 |
| | 0 | 0.964 | 0 | 0.48 | 10.5 | 0.005 | 28 | 29.2 |
| | 0.1 | 0.967 | 0 | 0.48 | 10.5 | 0.005 | 28 | 29.8 |
| | 0.2 | 0.969 | 0 | 0.48 | 10.5 | 0.005 | 28 | 30 |
| | 0 | 0.968 | 0 | 0.48 | 17.5 | 0.005 | 28 | 31.1 |
| | 0.1 | 0.97 | 0 | 0.48 | 17.5 | 0.005 | 28 | 31.5 |
| | 0.2 | 0.972 | 0 | 0.48 | 17.5 | 0.005 | 28 | 31.9 |
| | 0 | 0.971 | 0 | 0.48 | 24.5 | 0.005 | 28 | 30.1 |
| | 0.1 | 0.973 | 0 | 0.48 | 24.5 | 0.005 | 28 | 30.6 |
| | 0.2 | 0.975 | 0 | 0.48 | 24.5 | 0.005 | 28 | 30.9 |
| | 0 | 0.958 | 0 | 0.48 | 0 | 0.005 | 120 | 47.1 |
| | 0.1 | 0.96 | 0 | 0.48 | 0 | 0.005 | 120 | 49.7 |
| | 0.2 | 0.962 | 0 | 0.48 | 0 | 0.005 | 120 | 50.9 |
| | 0 | 0.964 | 0 | 0.48 | 10.5 | 0.005 | 120 | 60.3 |
| | 0.1 | 0.967 | 0 | 0.48 | 10.5 | 0.005 | 120 | 61.2 |
| | 0.2 | 0.969 | 0 | 0.48 | 10.5 | 0.005 | 120 | 61.4 |
| | 0 | 0.968 | 0 | 0.48 | 17.5 | 0.005 | 120 | 68.3 |
| | 0.1 | 0.97 | 0 | 0.48 | 17.5 | 0.005 | 120 | 68.8 |
| | 0.2 | 0.972 | 0 | 0.48 | 17.5 | 0.005 | 120 | 69.1 |
| | 0 | 0.971 | 0 | 0.48 | 24.5 | 0.005 | 120 | 64.1 |
| | 0.1 | 0.973 | 0 | 0.48 | 24.5 | 0.005 | 120 | 64.7 |

| | | | | | | | | |
|---|---|---|---|---|---|---|---|---|
| | 0.2 | 0.975 | 0 | 0.48 | 24.5 | 0.005 | 120 | 65.1 |
| [3] | 0 | 1.135 | 0 | 0.31 | 0 | 0.006 | 7 | 43.9 |
| | 0 | 1.134 | 0 | 0.31 | 5.2 | 0.008 | 7 | 45.5 |
| | 0 | 1.134 | 0 | 0.31 | 10.4 | 0.009 | 7 | 47.4 |
| | 0 | 1.133 | 0 | 0.31 | 15.6 | 0.011 | 7 | 50.4 |
| | 0.2 | 1.133 | 0 | 0.31 | 15.6 | 0.012 | 7 | 39.5 |
| | 0.1 | 1.133 | 0 | 0.31 | 15.6 | 0.012 | 7 | 51.4 |
| | 0.2 | 1.133 | 0 | 0.31 | 15.6 | 0.012 | 7 | 44.9 |
| [4] | 0 | 0.905 | 0 | 0.43 | 0 | 0.017 | 7 | 19.1 |
| | 0 | 0.905 | 0 | 0.43 | 31.5 | 0.024 | 7 | 35.6 |
| | 0.9 | 0.905 | 0 | 0.43 | 0 | 0.021 | 7 | 19.9 |
| | 0.9 | 0.905 | 0 | 0.43 | 31.5 | 0.025 | 7 | 38.1 |
| | 0 | 0.905 | 0 | 0.43 | 0 | 0.017 | 28 | 30 |
| | 0 | 0.905 | 0 | 0.43 | 31.5 | 0.024 | 28 | 49.2 |
| | 0.9 | 0.905 | 0 | 0.43 | 0 | 0.021 | 28 | 31.2 |
| | 0.9 | 0.905 | 0 | 0.43 | 31.5 | 0.025 | 28 | 52.3 |
| | 0 | 0.905 | 0 | 0.43 | 0 | 0.017 | 60 | 35.4 |
| | 0 | 0.905 | 0 | 0.43 | 31.5 | 0.024 | 60 | 56.1 |
| | 0.9 | 0.905 | 0 | 0.43 | 0 | 0.021 | 60 | 37 |
| | 0.9 | 0.905 | 0 | 0.43 | 31.5 | 0.025 | 60 | 58 |

CA/FA: ratio of coarse aggregate to fine aggregate, W/B, water to binder ratio, SP superplasticizer, B: binder (cement+nano-silica)

## References


1. Ashrafian, A.; Taheri Amiri, M.J.; Rezaie-Balf, M.; Ozbakkaloglu, T.; Lotfi-Omran, O. Prediction of compressive strength and ultrasonic pulse velocity of fiber reinforced concrete incorporating nano silica using heuristic regression methods. *Constr. Build. Mater.* **2018**, *190*, 479–494, https://doi.org/10.1016/j.conbuildmat.2018.09.047.
2. Salemi, N.; Behfarnia, K. Effect of nano-particles on durability of fiber-reinforced concrete pavement. *Constr. Build. Mater.* **2013**, *48*, 934–941, https://doi.org/10.1016/j.conbuildmat.2013.07.037.
3. Fallah, S.; Nematzadeh, M. Mechanical properties and durability of high-strength concrete containing macro-polymeric and polypropylene fibers with nano-silica and silica fume. *Constr. Build. Mater.* **2017**, *132*, 170–187, https://doi.org/10.1016/j.conbuildmat.2016.11.100.
4. Sadrmomtazi, A.; Fasihi, A. Influence of polypropylene fibers on the performance of nano-$sio_2$-incorporated mortar. *Iran. J. Sci. Technol. Trans. B-Eng.* **2010**, *34*, 385–395.
